# Accurate prediction of quantitative traits with failed SNP calls in canola and maize

**DOI:** 10.3389/fpls.2023.1221750

**Published:** 2023-10-23

**Authors:** Sven E. Weber, Harmeet Singh Chawla, Lennard Ehrig, Lee T. Hickey, Matthias Frisch, Rod J. Snowdon

**Affiliations:** ^1^ Department of Plant Breeding, Justus Liebig University, Giessen, Germany; ^2^ Department of Plant Science, University of Manitoba, Winnipeg, MB, Canada; ^3^ Centre for Crop Science, Queensland Alliance for Agriculture and Food Innovation, The University of Queensland, St Lucia, QLD, Australia; ^4^ Department of Biometry and Population Genetics, Justus Liebig University, Giessen, Germany

**Keywords:** genomic selection, genome structural variants, presence-absence variations, machine learning, SNP markers

## Abstract

In modern plant breeding, genomic selection is becoming the gold standard to select superior genotypes in large breeding populations that are only partially phenotyped. Many breeding programs commonly rely on single-nucleotide polymorphism (SNP) markers to capture genome-wide data for selection candidates. For this purpose, SNP arrays with moderate to high marker density represent a robust and cost-effective tool to generate reproducible, easy-to-handle, high-throughput genotype data from large-scale breeding populations. However, SNP arrays are prone to technical errors that lead to failed allele calls. To overcome this problem, failed calls are often imputed, based on the assumption that failed SNP calls are purely technical. However, this ignores the biological causes for failed calls—for example: deletions—and there is increasing evidence that gene presence–absence and other kinds of genome structural variants can play a role in phenotypic expression. Because deletions are frequently not in linkage disequilibrium with their flanking SNPs, permutation of missing SNP calls can potentially obscure valuable marker–trait associations. In this study, we analyze published datasets for canola and maize using four parametric and two machine learning models and demonstrate that failed allele calls in genomic prediction are highly predictive for important agronomic traits. We present two statistical pipelines, based on population structure and linkage disequilibrium, that enable the filtering of failed SNP calls that are likely caused by biological reasons. For the population and trait examined, prediction accuracy based on these filtered failed allele calls was competitive to standard SNP-based prediction, underlying the potential value of missing data in genomic prediction approaches. The combination of SNPs with all failed allele calls or the filtered allele calls did not outperform predictions with only SNP-based prediction due to redundancy in genomic relationship estimates.

## 1 Introduction

Genomic prediction has become the gold standard to identify genetically superior accessions within breeding materials. Henderson (1975) was among the first breeders to use relatedness based on pedigree information, along with phenotypic data, for breeding value prediction in a mixed linear model framework. Based on recent advances in genome sequencing technologies, genomic data is used today to replace pedigree relationships in statistical prediction models (Bernardo, 1994; Meuwissen et al., 2001; VanRaden, 2008). The increasingly accurate genome sequencing today allows the identification of millions of polymorphisms across the genome with high quality and confidence. Along with phenotypic measurements, these genotypic profiles can be used to predict the breeding values of non-phenotyped individuals with statistical models (Lande and Thompson, 1990; Meuwissen et al., 2001; VanRaden, 2008). These statistical methods utilize phenotypic and genotypic information from some genotypes (training population) to predict genotypes with only genotypic information. Over the years, several mathematical models have been proposed for genomic prediction; the commonly used models include GBLUP (Bernardo, 1994; Meuwissen et al., 2001; VanRaden, 2008), Reproducing Kernel Hill Regression (RKHS) (de los Campos et al., 2009), and models from the Bayesian alphabet like Bayesian LASSO (Park and Casella, 2008) or Bayesian ridge regression (Pérez and de los Campos, 2014). These models differ in their assumption of variance components, marker effects, marker modes of action, and model assumptions. More recently, machine learning algorithms have also been implemented for genomic prediction (Azodi et al., 2019; Pérez-Enciso and Zingaretti, 2019).

Genotypic information utilized for genomic prediction normally comprises biallelic single-nucleotide polymorphisms (SNPs) that are enormously abundant in eukaryotic genomes (Rafalski, 2002; Frazer et al., 2007; Ganal et al., 2009). Besides their high frequency, SNPs are not always able to explain all of the genetic variations, particularly for more complex traits, which tend to be characterized by “missing heritability” (Manolio et al., 2009; Forer et al., 2010). Genome structural variants (SV) are another type of genomic polymorphism that might explain some of this missing heritability (Manolio et al., 2009; Génin, 2020; Theunissen et al., 2020; Zhou et al., 2022). Plant genomes exhibit widespread SV including copy number variations, deletions, or insertions (Eichten et al., 2011; Fuentes et al., 2019; Gabur et al., 2019; Schiessl et al., 2019; Yang et al., 2019; Chawla et al., 2021), and because these are not always in linkage disequilibrium with neighboring SNPs, their effects are not always captured by the surrounding SNP variants (Gabur et al., 2018). However, such polymorphisms have been shown to be associated with a wide range of agronomical important traits (Gabur et al., 2018; Vollrath et al., 2021a; Vollrath et al., 2021b; Yuan et al., 2021). Specifically, it was shown that SVs are associated with disease resistance and flowering time in canola (Gabur et al., 2018; Gabur et al., 2020; Vollrath et al., 2021a; Vollrath et al., 2021b), disease resistance and boron toxicity tolerance in barley (Sutton et al., 2007; Muñoz-Amatriaín et al., 2013), pathogen response and aluminum tolerance in maize (Beló et al., 2010; Maron et al., 2013), and plant height and heading date in wheat (Li et al., 2012; Nishida et al., 2013), for example [for a comprehensive review, see Gabur et al. (2019)].

In large-scale breeding populations, SNPs are usually assessed with SNP arrays; however, these platforms are prone to technical errors that result in failed allele calls. Markers with a very high failed call rate are commonly discarded from downstream genetic analyses (Zhao et al., 2013; Lehermeier et al., 2014; Werner et al., 2018a; Knoch et al., 2021). For the remaining markers, failed allele calls need to be imputed to avoid large numbers of missing data points for further genetic studies. There are numerous methods to impute missing allele calls, with the simplest being the population mean/median (Endelman, 2011; Covarrubias-Pazaran, 2016; Covarrubias-Pazaran, 2018) or more advanced algorithms like “BEAGLE”, “SHAPEIT”, and “IMPUTE2” (Browning and Browning, 2007; Howie et al., 2011; Delaneau et al., 2012; Browning et al., 2018) which rely on allele frequencies, haplotypes, and flanking marker information. Regardless of the approach, imputation assumes that each missing marker call represents a genuine technical error. However, using whole-genome sequencing and patterns of inheritance in structured populations, Gabur et al. (2018) have demonstrated that, in complex crop genomes, missing allele calls can often be caused by polymorphic presence–absence variations resulting from deletions of sequences spanning SNP loci. Omitting or imputing failed allele calls can hence obscure valuable marker–trait associations. Commonly, SNPs with excessive failed calls are frequently eliminated from new iterations of genotyping arrays because they are considered technically unreliable (Boichard et al., 2012; Bayer et al., 2017). This can lead to considerable loss of potentially important genotype information and false imputations.

Whole-genome long-read sequencing data can be used to accurately identify structural variants (Francia et al., 2015; Dumschott et al., 2020; Chawla et al., 2021), enabling the validation of presence–absence variations detected in SNP array data (Gabur et al., 2018). However, genotyping a whole breeding population with thousands of genotypes *via* whole-genome long-read sequencing is economically not feasible. Targeted long-read sequencing of agronomically interesting genomic regions using ReadUntil (Edwards et al., 2019) might provide an alternative, which is a financially viable approach to identify genome structural variations at the population scale. However, application at scale in a breeding program may still be challenging. Furthermore, SNP arrays are well established as one of the main methods of choice for breeders to genotype their populations, hence the detection of presence–absence variations using these arrays comes at no additional cost. Most published work, to date, linking structural variants to quantitative traits have focused on association studies (see Gabur et al. (2019) for a detailed review). Only few studies have investigated their use for genomic prediction (Hay et al., 2018; Lyra et al., 2019; Chen et al., 2021; Knoch et al., 2021; Lamb et al., 2021), most of which utilize structural variants called from long- or short-read sequencing data. The aim of this study was to examine the value of potential presence–absence variants in the form of failed allele calls from SNP arrays in genomic predictions. To our knowledge, previously, this has only been done in association studies (Gabur et al., 2018; Gabur et al., 2020; Vollrath et al., 2021a; Vollrath et al., 2021b), making this the first attempt to utilize failed allele calls in genomic prediction. Specifically, the following questions were addressed: (1) How predictive are failed allele calls in genomic prediction and (2) can the addition of failed allele call information to standard SNPs improve genomic prediction accuracy? To answer these questions, published datasets from maize and canola were utilized for genomic predictions based on failed allele calls and genome-wide SNP markers, respectively. Prediction accuracy from cross-validation was subsequently used to assess marker–trait associations. Genomic prediction was performed with GBLUP, Bayesian LASSO, EGBLUP, RKHS, and Gradient Boosting and Support Vector Machines. Furthermore, two naive methods were developed and deployed to select failed allele calls based on population information. Using failed allele calls as indicators for presence–absence events, we show that these are as predictive as standard SNP markers for agronomic traits, underlining the potential information content of missing data in SNP arrays.

## 2 Materials and methods

### 2.1 Datasets

Two previously published datasets were examined in this study. The first was a canola dataset from a spring-type canola hybrid breeding program (Jan et al., 2016). Here two male sterile lines were crossed to 475 doubled-haploid (DH) pollinators to create 950 test crosses. The test crosses were subsequently tested for seed yield, flowering time, field emergence, lodging, oil content, oil yield, and glucosinolate content in a multi-environment trial at four different locations in 2 years. All parental lines were genotyped with the Illumina *Brassica* 60 k SNP array (Clarke et al., 2016). In total, 910 test crosses with complete phenotypic and genotypic records are available. The phenotypic data was published on an adjusted trait mean per genotype.

The second dataset represent two nested association mapping (NAM) populations of Flint and Dent maize. The population consists of 10 Dent and 11 Flint half-sib DH families. The lines were evaluated as test crosses, the DH Dent lines were all crossed to a single Flint tester line (UH007), and all DH Flint lines were crossed to a single Dent tester (F353). All DH lines were genotyped with the Illumina MaizeSNP50 SNP array (Ganal et al., 2011). This population was first described in Bauer et al. (2013), while Lehermeier et al. (2014) published phenotypic data from four locations for the Dent panel and at six locations for the Flint panel, including dry matter yield (DMY), dry matter content (DMC), plant height (PH), days till tasseling (DtTAS), and days till silking (DtSILK). The published field data was adjusted independently in the Flint and Dent pool, following the methods of the original publication. In total, complete phenotypic and genotypic data were available for test crosses from 847 Dent maternal lines and 918 Flint maternal lines.

### 2.2 Genotypic data

SNP matrices were filtered to remove markers with non-unique positions (multiple BLASTn hits of flanking sequences) on reference genomes. In canola, we utilized the *Brassica napus* Express 617 genome v2 (Lee et al., 2020) and in maize the B73 AGPv2 genome (Schnable et al., 2009). The genotypic data for the two maize pools was filtered jointly as one population. Compared to standard filtering pipelines, which removed SNPs with a certain proportion of failed calls, we treated failed SNP calls as third allele. In the first step, the coding for the original marker matrix was A/A, A/B, B/B, and F/F (“homozygous missing/failed allele”). Consequently, in this set, the markers were filtered according to an expected ≥0.095 (treating F as third allele), which corresponds to a minor allele frequency ≥0.05 in a biallelic case. From that, two copies of this matrix were created, one corresponding to the standard SNPs and one corresponding to the failed allele calls.

The copy corresponding to standard SNPs was then phased and imputed with the software “BEAGLE V5.2” (Browning and Browning, 2007; Browning et al., 2018). Subsequently, the markers were filtered for minor allele frequency ≥0.05 (to rule out monomorphic markers which could arise after imputation) and converted into numeric format (0, 1, 2 for A/A, A/B, and B/B).

The copy corresponding to the failed allele calls was recoded to successful call/successful call (regardless of allelic state) and F/F (“homozygous missing/failed allele”). This matrix was then also filtered for minor allele frequency ≥0.05 (to rule out monomorphic markers) and then converted into numeric format (0, 2 for successful call/successful call and F/F).

For canola, the processing resulted in 31,085 markers with successful allele calls and 7,169 markers with failed allele calls. In maize, we obtained 39,624 markers with successful allele calls and 8,024 markers with failed allele calls.

### 2.3 Population structure

For both datasets, the population structure was assessed by calculating the Euclidean distance between genotypes based on standard SNP markers and failed allele calls, respectively. Subsequently, the genotypes of each species were clustered into two subpopulations each using k-means clustering. A principal component analysis based on the genetic distance was conducted, and the first two principal components were utilized to visualize population stratification.

### 2.4 Methods to filter failed SNP calls with biological reasons

In the following two sections, we introduce two pipelines designed to distinguish between random failed allele calls and non-random systematic failed allele calls. This is done to strengthen the confidence that those failed allele calls stem from some biological reason, which hinders an allele call. These pipelines only rely on population measures and statistical tests.

#### 2.4.1 Pool specificity

An important step in hybrid breeding is the creation of distinct genetic pools. Hence, the datasets assessed in this study naturally show a strong population structure corresponding to divergent genetic pools. In such populations, a proportion of alleles become pool-specific due to selection and genetic drift. On the other hand, technical errors can, by definition, not be pool specific; hence, they cannot show a bias between two different hybrid breeding pools. We thus assumed that there should be no relationship between subpopulation assignment and SNP call failure. In the breeding populations examined here, the populations for each species investigated split into two major gene pools. Hence, we expect that technical errors and successful allele calls should distribute equally in the two subpopulations. A $\chi^{2}$ test of independence was utilized to test if there is an influence of subpopulation on allele call or failure. Pool assignment was based on k-means clustering with standard SNPs. Specifically, we tested for each failed allele call as follows:

• *H0*: failed allele call *versus* successful marker call and pool assignment is not related in the populations.
• *H1*: failed allele call *versus* successful marker call and pool assignment is related in the populations.

When *H0* is rejected, this is considered to be biological evidence for pool specificity of marker failure rather than a technical failure. Hence, we filter this failed allele call marker from the set of all failed allele calls and use it further in prediction models. After adjustment according to Benjamini and Hochberg (1995), the *p*-values were compared at a threshold of *α* = 0.05.

#### 2.4.2 Linkage disequilibrium

Linkage disequilibrium (LD) between markers on the same chromosome was calculated as *r^2^* (Hill and Robertson, 1968) in “SelectionTools” (http://population-genetics.uni-giessen.de/~software/), treating each failed allele call as an independent marker with the same genome position as its corresponding standard SNP.

If a failed marker call is purely due to a technical error, the failed call should not be in LD with any other marker. If the failed call is in considerable LD with markers on the same chromosome, we can assume that the failure is inherited together with other markers and the failure has a biological reason. Subsequently, a simple Student’s *t*-test can be used to compare the LD patterns. If the LD of the failed marker with all other standard SNP calls on the same chromosome is considerably lower than its standard SNP counterpart, we can assume that the failure is due to a technical error. Specifically, for each failed marker call, we test the following hypotheses:

• *H0*: failed allele call and successful marker call show the same average LD to all standard markers on the same chromosome.
• *H1*: failed allele call and successful marker call show lower average LD to all standard markers on the same chromosome.

When *H0* is failed to reject, failed allele calls are considered to be in LD to markers on the same chromosome. Hence, we filter this failed allele call marker from the set of all failed allele calls and use it further in prediction models. After adjustment according to Benjamini and Hochberg (1995), the *p*-values were compared at a threshold of *α* = 0.05.

### 2.5 Genomic prediction models

Six genomic prediction models were used to predict test cross performance. Two variations of GBLUP, two Bayesian methods, and two machine learning methods were used, covering parametric and non-parametric models. We applied standard GBLUP and extended GBLUP (EGBLUP) to account for second-order additive*additive epistasis (Jiang and Reif, 2015). Furthermore, we used the Bayesian LASSO model (Park and Casella, 2008) due to its capability for marker-specific shrinkage and the semiparametric model RKHS for modeling of higher-order epistasis (de los Campos et al., 2009). These approaches were complemented by the machine learning algorithms gradient boosting (Friedman, 2001) and support vector machines (SVM) (Boser et al., 1992).

In GBLUP and EGBLUP, the underlying mixed linear model is:


$$y=X\beta +Z_{a}a+Z_{i}i+e$$

where y is the vector of observations for a trait under consideration,  β is the vector of fixed effects, a is the vector of random additive marker effects, i is the vector of random epistatic effects, and e is the random residual term. $Z_{a}$ and $Z_{i}$ are design matrices relating the random effects to the phenotypic records. X is the design matrix for fixed effects and, in the case of the canola dataset, a column of ones modeling the intercept and an additional column for the male sterile mother. In the maize datasets, X has a column of ones for the intercept and an additional 10 (Dent dataset) or 11 (Flint dataset) columns that assign individuals to half-sib families.

It is assumed that $a∼N\left(0,G_{a}\sigma_{a}^{2}\right),\;i∼N\left(0,G_{aa}\sigma_{aa}^{2}\right)\;\text{and}\;e∼N\left(0,I\sigma_{e}^{2}\right),\;$ where $\sigma_{a}^{2},\;\sigma_{aa}^{2}$ , and $\sigma_{e}^{2}$ are additive genetic variance, epistatic genetic variance, and error variance, respectively. $G_{a}$ and $G_{aa}$ are the respective additive and epistatic relationship matrices, and I is an identity matrix. Depending on the inclusion of epistatic effects, the corresponding terms were included or omitted.

The additive genomic relationship matrix was calculated following VanRaden (2008):


$$G=\frac{ZZ'}{2\sum^{}p_{i}\left(1-p_{i}\right)}$$

In the case of prediction based on standard SNPs, the elements of Z are represented by (0-2p_i_) for homozygous allele A, (1-2p_i_) for the heterozygous state, and (2-2p_i_) for homozygous allele B, with p_i_ being the allele frequency of the B allele. For prediction based on all failed calls or filtered failed allele calls, the elements of Z are represented by (0-2p_i_) for successful allele calls and (2-2p_i_) for failed allele calls, with p_i_ being the allele frequency of the failed allele call. Furthermore, the combination of (i) SNPs and failed allele calls, (ii) SNPs and failed allele calls filtered by pool specificity, and (iii) SNPs and failed allele calls filtered by LD were considered.

A second-order (additive*additive) epistatic relationship matrix can be approximated with $G_{aa}=G\#G$ , where # denotes the pointwise (Hadamard) product operation (Henderson, 1985; Jiang and Reif, 2015).

All the mixed linear models described in this section were implemented and solved with the r package “sommer” (Covarrubias-Pazaran, 2016; Covarrubias-Pazaran, 2018), which also computes all model parameters including variance components.

The formula describing the Bayesian LASSO model, following Park and Casella (2008), is:


y=Xβ+Ma+e

where y is the vector of observations for a trait under consideration, β is the vector of fixed non-genetic effects, a is the vector of additive effects, X is the design matrix as described in the GBLUP section, and M is the incidence matrix relating phenotypic records with the respective marker. In standard SNP-based predictions, the elements of M are 0 for homozygous allele A, 1 for heterozygous, and 2 for homozygous allele B. In the case of prediction based on failed or filtered failed allele calls, the elements of M are 0 for a successful allele call and 2 for the failed allele call. Furthermore, we also considered the combination of (i) SNPs and failed allele calls, (ii) SNPs and failed allele calls filtered by pool specificity, and (iii) SNPs and failed allele calls filtered by LD. The coefficients of the fixed ( β ) effects are assigned flat priors, and the coefficients of the marker effects ( a ) are assigned double-exponential priors. This allows the shrinkage of some marker effects to effectively zero, introducing sparsity into the model. This model allows a stronger shrinkage of the marker effects, which may be useful especially for technical errors. Here e is the random residual term. This model was conducted in the r software with the package “BGLR” (Pérez and de los Campos, 2014), which computes all the model parameters. Default settings were utilized.

Following de los Campos et al. (2009) with kernel averaging, the RKHS model has the following form:


$$y=X\beta +\sum_{l=1}^{L}u_{l}+e$$

with


$$p\left(\beta ,u_{1},\ldots u_{L},e\right)\propto \;\prod_{l=1}^{L}N\left(u|0,\;K_{ul}\sigma_{ul}^{2}\right)\;N(e|0,\;I\sigma_{e}^{2})\;$$

where y is the vector of observations, while $K_{ul}$ represents an n*n kernel calculated based on the Euclidean distance between genotypes called (a) standard SNPs, (b) failed allele calls, (c) failed allele calls filtered by pool specificity, and (d) failed allele calls filtered by LD or a combination of (a) with (b), (c), or (d). The kernel was chosen to be a Gaussian kernel with the *l*th value of the bandwidth parameter {0.1, 0.5, 2.5}. Xβ is treated in a similar manner to the Bayesian LASSO, and $u_{l}$ is assumed to be random. That way, the different random effects, i.e., the three kernel matrices from the three bandwidth parameters, are weighted by their variance components. Again, e is the random residual term. This model was also conducted in the r software with the package “BGLR” (Pérez and de los Campos, 2014), which computes all the model parameters using the default setting of the package.

Gradient boosting sequentially builds ensembles of decision trees. The algorithm starts with an intercept estimation. Subsequently, it sequentially fits models on the residual of its predecessor (Friedman, 2001). The goal of each model is to minimize the prediction error of the previous model. Generally, the model can be described with following formula:


$$y=1\mu +\;\sum_{m=1}^{M}\eta f_{m}\left(X\right)+e$$

where y is the vector of observations, μ is the overall intercept, and f is the base learning function, i.e., a decision tree. η is a shrinkage parameter, controlling the overall contribution of each decision tree to the total prediction. X is a matrix of (a) standard SNPs, (b) failed allele calls, (c) failed allele calls filtered by pool specificity, and (d) failed allele calls filtered by LD or a combination of (a) with (b), (c), or (d). Furthermore, in the case of the canola dataset, an additional column for the male sterile mother was added. In the maize datasets, an additional 10 (Dent) or 11 (Flint) columns were added that assign individuals to half-sib families. This model was conducted with the r package “xgboost” (Chen and Guestrin, 2016). Hyperparameters “eta”, “gamma”, “max_depth”, “min_child_weight”, “subsample”, and “colsample_bytree” were optimized *via* Bayesian hyperparameter optimization using the r package “rBayesianOptimization” (Yan, 2022).

The SVM model performs a form of nonlinear regression; specifically, the ϵ-support vector regression (Chang and Lin, 2011) is utilized. It performs non-linear regression by projecting the data into higher dimensional space with a kernel function. This model was conducted with the r package “kernlab” (Karatzoglou et al., 2004), using the radial basis function as kernel function. Hyperparameters epsilon and cost were optimized with Bayesian hyperparameter optimization using the r package “rBayesianOptimization” (Yan, 2022). Prediction was based on the matrix of (a) standard SNPs, (b) failed allele calls, (c) failed allele calls filtered by pool specificity, and (d) failed allele calls filtered by LD or a combination of (a) with (b), (c), or (d). Furthermore, in the case of the canola dataset, an additional column was added for the male sterile maternal line, whereas for maize an additional 10 (Dent dataset) or 11 (Flint dataset) columns were added, which assign individuals to half-sib families.

### 2.6 Evaluation of prediction accuracy

The prediction accuracy for the two datasets was evaluated using fivefold cross-validation. The population was randomly divided into five equal-sized sets. In each fold, the prediction models were trained on four sets (training population), and then these trained models were utilized to predict the remaining set (validation population) with masked phenotypic data. This process was repeated until each set served as the validation population once. The accuracy was measured using the Pearson correlation coefficient (*r*) between the observed and predicted phenotypic values of the validation set in each fold. To ensure robustness, this entire procedure was repeated 30 times.

### 2.7 Genomic relationship

To assess how well relationship based on standard SNPs is also captured by one of the failed allele call marker sets, we used the relationship coefficients obtained from the relationship matrix calculated following VanRaden (2008) (see above) and calculated the Pearson correlation between relationship coefficients from SNPs and those from the failed allele calls.

### 2.8 Simulation

To test how high prediction accuracy with failed allele calls can get by chance, i.e., random association between failed calls (due to random technical problems of the array), a simulation was conducted. The basis of the simulation was the genotypic data described in Section 2.2. Here we took the imputed marker matrices as “true” genotypic data and simulated marker effects. In total, 100, 1,000, and 10,000 markers were sampled to serve as QTL. Subsequently, marker effects were sampled from a normal distribution with mean = 0 and variance = 1. The phenotype was then obtained by adding a random residual term to the total additive value of the individual. The residuals were sampled from a normal distribution with mean = 0 and variance = $V_{e}$ . $V_{e}$ was calculated as $\frac{V_{g}}{H^{2}}-V_{g}$ , where $V_{g}$ is the total genetic variance, i.e., variance of the breeding values, and $H^{2}$ is the heritability calculated as $H^{2}=\frac{V_{g}}{V_{g}+V_{e}}$ . Three heritabilities ( $H^{2}$ = 0.4, 0.6, and 0.8) were simulated for each number of QTL.

According to the number of failed calls observed before imputation, 658,730 entries of the marker matrix in canola and 3,712,821 entries of the marker matrix in maize were randomly sampled to be failed calls and treated as described in Sections 2.2. and 2.4. In each simulation, genomic prediction was conducted with the GBLUP model based on SNPs and failed allele calls. Prediction accuracy was then measured with fivefold cross-validation with 10 repetitions (see Section 2.6). For each combination of number of QTL and heritability, 100 simulations were conducted to obtain a robust result.

## 3 Results

### 3.1 Canola

**Figure 1:**
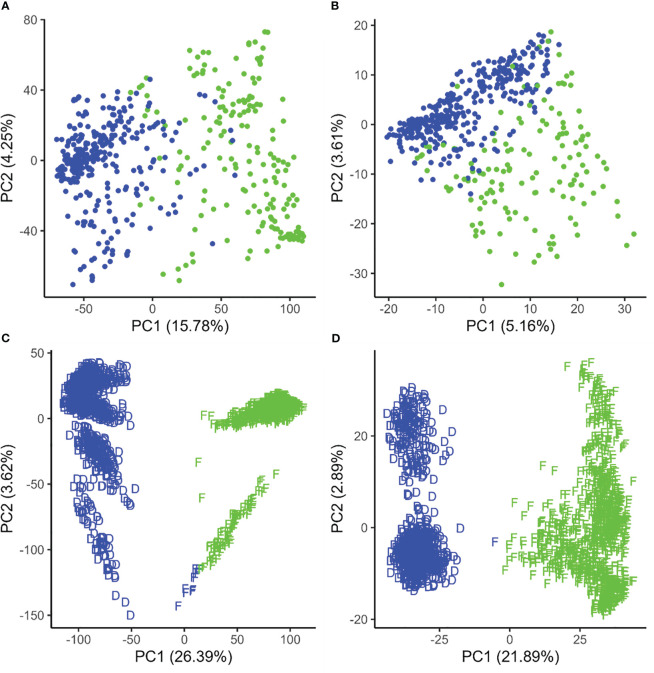
Population structure displayed by the first two principal components of the genetic distance in canola **(A, B)** and maize **(C, D)** based on standard single-nucleotide polymorphisms **(A, C)** and failed allele calls **(B, D)**. The colors (red and blue) represent clusters based on k-means clustering, while the D and F shapes in maize represent true Dent and Flint clusters.

In canola, k-means clustering based on standard SNP markers revealed a considerable population stratification into two subpopulations/pools which we designated as pool A and pool B, respectively (**Figure 1**). The lines in pool A had, on average, 686.80 (median = 618.5) failed allele calls, while the lines in pool B had, on average, 848.21 (median = 767) failed allele calls (**Supplementary Figure S1**). The first three principal components based on standard SNPs together explain 23.25% of the variance in the marker data. On the other hand, the population structure based on failed allele calls also shows a distinction into two subpopulations based on k-means clustering; however, clustering did not result in the same subpopulation assignment compared to the standard SNPs (**Figure 1**). Here the first three principal components together explain 10.56% of the variance in the failed marker set. A visual inspection of the first two components of the two respective marker sets show a considerable overlap of the subpopulations.

Each possible failed allele call was tested for pool specificity. In canola, 1,989 failed allele calls showed significant pool specificity. The lines in pool A carry, on average, 302.26 (median = 283) pool-specific failed allele calls, and the lines in pool B carry, on average, 398.93 (median = 409) (**Supplementary Figure S1**). The LD of each possible failed allele call was compared to its standard SNP counterpart in both datasets. This resulted in 1,084 failed allele calls showing considerable LD with standard SNPs on the same chromosome. The lines in pool A carry, on average, 206.72 (median = 202) failed allele calls filtered by LD, while the lines in pool B carry 274.77 (median = 301) failed allele calls on average (**Supplementary Figure S1**). Subsequently, the markers filtered by the two methods described were utilized for the following analysis. Combining SNPs and all failed allele calls yields a total of 38,254 markers. When SNPs are combined with failed allele calls filtered by pool specificity, there are 33,074 markers. The combination of SNPs with failed allele calls filtered by LD results in a set of 32,169 markers.

**Figure 2:**
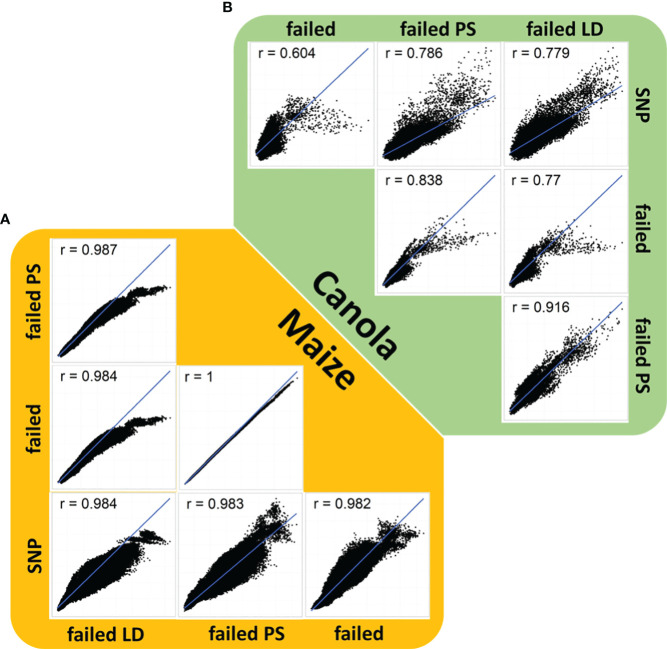
Correlation plot of genomic relationship coefficients based on single-nucleotide polymorphisms, failed allele calls (failed), failed allele calls filtered by pool specificity (failed PS), and failed allele calls filtered by LD (failed LD) in **(A)** canola (green) and **(B)** maize (orange).

An analysis of genomic relationships showed a high correspondence between the estimates of relationship based on standard SNPs, failed allele calls, and the two filtering methods (**Figure 2**). Correlations between the relationships based on SNPs and the three failed allele call sets were generally high in canola (**Figure 2**). The lowest correlation (*r* = 0.604) was observed between the SNP-based relationship and the relationship based on failed alleles (**Figure 2**). In contrast, stronger correlations were found between the SNP-based relationships and the failed allele calls filtered by pool specificity (0.786) or the failed allele calls filtered by LD (0.779), respectively (**Figure 2**).

**Figure 3:**
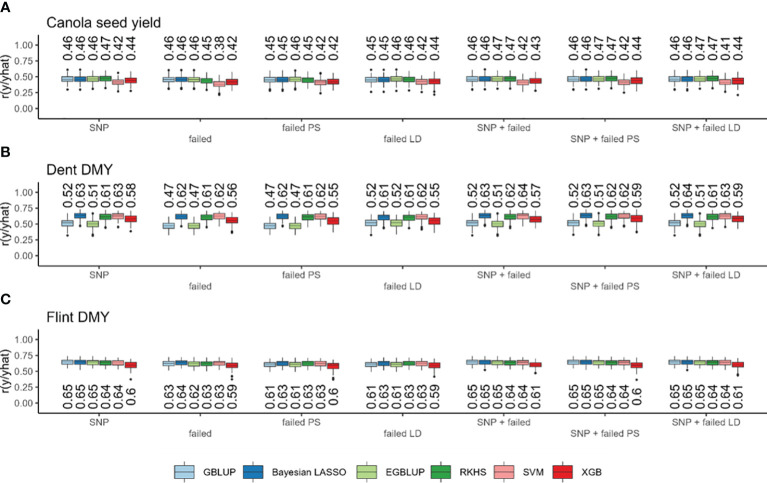
Prediction accuracy (*r*) based on standard single-nucleotide polymorphisms (SNPs), failed SNP calls (failed), failed SNP calls filtered by pool specificity (failed PS), and failed SNP calls filtered by LD (failed LD) as well as their combination with GBLUP (light blue), Bayesian Lasso (dark blue), EGBLUP (light green), RKHS (dark green), SVM (pink), and XGB (red). In canola seed yield **(A)**, maize Dent dry matter yield **(B)** and maize Flint dry matter yield **(C)**. Values above the boxplots represent median values across all cross-validation runs.

**Figure 4:**
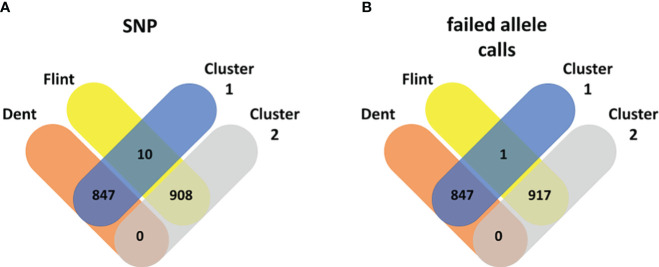
Venn diagram: Maize pool assignment to Dent (red) and Flint (yellow) subpools vs. pool assignment based on k-means clustering into cluster 1 (blue) and cluster 2 (gray) based on the genetic distance from standard single-nucleotide polymorphisms **(A)** and failed allele calls **(B)**.

Genomic prediction based on standard SNPs resulted in prediction accuracies ranging from 0.174 with SVM for field emergence to 0.813 with XGB for oil content (**Supplementary Figure S2**). Considerable differences could be observed between traits, while the differences between marker sets or prediction models were only very small (**Figure 3**; **Supplementary Figure S2**). Only in the trait field emergence did all other models considerably outperformed the two machine learning models SVM and XGB (**Supplementary Figure S2**). Across all models with standard SNPs, the prediction accuracy was lowest for field emergence, followed by lodging, seed yield, glucosinolate content, days to flowering, oil yield, and oil content (**Figure 4**; **Supplementary Figure S2**). The prediction accuracy based on failed allele calls was generally similar to the accuracy of standard SNP-based predictions for all traits (**Figure 3**; **Supplementary Figure S2**). When using markers from one of the methods to filter failed allele calls, the prediction accuracy did not improve compared to the prediction based on all failed allele calls. However, we also observed no further decrease in prediction accuracy (**Figure 3**; **Supplementary Figure S2**). When combining both (i) SNPs and failed allele calls, (ii) SNPs and failed allele calls filtered by pool specificity, and (iii) SNPs and failed allele calls filtered by LD, genomic prediction did not change compared to standard SNP-based prediction (**Figure 3**; **Supplementary Figure S2**).

### 3.2 Maize

In maize, k-means clustering based on standard SNP markers revealed a strong population stratification into two major groups that more or less correspond to the respective Flint and Dent pools (**Figure 1**). The lines in the Dent pool had, on average, 1,796.76 (median = 1,756) failed allele calls, while the lines in the Flint pool had on average 2,088.72 (median = 2,100) failed allele calls (**Supplementary Figure S1**). k-means clustering based on standard SNP markers assigned 10 genotypes of the Flint pool wrongly to the Dent pool (**Figures 1**, **3**). Here the first three principal components together explain 33.03% of the variance in the marker data. The population structure based on failed allele calls also shows a strong distinction into two subpopulations. Clustering based on failed allele calls assigned only one genotype of the Flint pool incorrectly to the Dent pool (**Figures 1**, **4**). The first three principal components cumulatively explain 27.38% of the variance in the failed marker set. A visual inspection of the first two principal components of the two respective marker sets did not show any overlap between the Flint and Dent pools (**Figure 1**). Further subclusters could be seen in both the Flint and Dent pools which likely correspond to different families of the NAM population within the Dent and Flint material (**Figure 1**).

Testing each possible failed allele call for pool specificity showed that 7,286 markers with failed allele calls show pool specificity. The lines in the Dent pool carry, on average, 1,647.95 (median = 1,614) pool-specific failed allele calls, whereas the lines in the Flint pool carry, on average, 1,962.51 (median = 1,996) (**Supplementary Figure S1**). The LD-based method, on the other hand, filtered 2,156 failed allele calls that show considerable LD with standard SNPs on the same chromosome. Here the lines in the Dent pool carry, on average, 650.34 (median = 661) failed allele calls filtered by LD, while the lines in the Flint pool carry, on average, 913.88 (median = 949) (**Supplementary Figure S1**). Subsequently, the markers filtered by these two methods were utilized for the following analysis. The combination of SNPs and all failed allele calls yields a total of 47,648 markers. When we merge SNPs with failed allele calls filtered by pool specificity, there are 46,910 markers. Meanwhile, the combination of SNPs with failed allele calls filtered by LD produces a set of 41,780 markers.

An analysis of genomic relationships in maize showed high correlations between estimates of relationship based on standard SNPs, failed allele calls, and the two filtering methods (**Figure 2**). In maize, the lowest correlation (*r* = 0.982) detected was observed between the SNP-based relationship and failed allele calls (**Figure 2**). However, the difference to the correlations between standard SNPs and failed allele calls filtered by pool specificity (*r* = 0.984) or failed allele calls filtered by LD (*r* = 0.983) was considerably lower than the corresponding differences in canola (**Figure 2**). In all correlation plots of relationhip estimates, there were observable clusters corresponding to the strong distinction into genetically distinct pools (**Figure 2**).

#### 3.2.1 Dent pool

Within the maize Dent pool, genomic prediction based on standard SNPs resulted in prediction accuracies in the range from 0.505 with EGBLUP for DMY to 0.850 with SVM for DMC. There were considerable differences between traits and models, while the differences between marker sets were only very small. With standard SNPs, the prediction accuracy across all models was lowest for DMY, followed by PH, DtSILK, DtTAS, and DMC (**Figure 3**; **Supplementary Figure S3**). Interestingly, GBLUP, EGBLUP, and XGB showed lower prediction accuracies compared to all other models across all traits, with the exception of PH (**Figure 3**; **Supplementary Figure S3**), for which XGB showed slightly higher prediction accuracies than GBLUP and EGBLUP (**Supplementary Figure S3**). Across traits, there was no consistent ranking between the remaining models Bayesian LASSO, RKHS, and SVM, with Bayesian LASSO yielding the highest prediction accuracy for DMY, PH, and DtTAS, whereas SVM yielded the highest prediction accuracy for DMC and DtSILK. Using all failed allele calls reduced the prediction accuracy only marginally, while the two alternative methods to filter failed allele calls gave a similar prediction accuracy compared to the use of all failed allele calls (**Figure 3**; **Supplementary Figure S3**). The combination of both (i) SNPs and failed allele calls, (ii) SNPs and failed allele calls filtered by pool specificity, and (iii) SNPs and failed allele calls filtered by LD in genomic prediction did not change the prediction accuracy compared to standard SNP-based prediction (**Figure 3**; **Supplementary Figure S3**).

#### 3.2.2 Flint pool

Within the maize Flint pool, genomic prediction based on standard SNPs resulted in prediction accuracies in the range from 0.598 with XGB for DMY to 0.909 with GBLUP for DtSILK (**Figure 3**; **Supplementary Figure S3**). There were considerable differences again between traits and models. The differences between marker sets were only very small (**Figure 3**; **Supplementary Figure S4**). Across all models, the prediction accuracy based on standard SNPs was the lowest for DMY, followed by PH, DtSILK, DtTAS, and DMC (**Figure 3**; **Supplementary Figure S4**). Generally, the prediction accuracies obtained from XGB were among the worst across all traits, while GBLUP and EGBLUP showed considerably lower prediction accuracies only for DtTAS and PH (**Figure 3**; **Supplementary Figure S4**). Generally, the differences between models were much smaller in scale than the differences in prediction accuracy between traits (**Figure 3**; **Supplementary Figure S3**). The prediction based on failed allele calls reduced the prediction accuracy again only marginally. The two methods to filter failed allele calls did not improve the prediction accuracy compared to the prediction based on all failed allele calls. However, no large decrease in prediction accuracy could be observed. Combining both (i) SNPs and failed allele calls, (ii) SNPs and failed allele calls filtered by pool specificity, and (iii) SNPs and failed allele calls filtered by LD in genomic prediction did not change the prediction accuracy compared to standard SNP-based prediction (**Figure 3**; **Supplementary Figure S3**).

### 3.3 Simulation

Applying the filtering methods to the random failed allele calls within each simulation repetition only rarely yielded any failed allele call after filtering. If failed allele calls were left in the simulations, there were only up to two failed allele calls left after filtering. Consequently, we applied genomic prediction only with the complete set of failed allele calls in each simulation. Generally, the prediction accuracies based on SNPs for all simulated traits in both crops followed closely the simulated heritability, independent of the number of QTL. With failed allele calls, on the other hand, the prediction accuracy was close to zero across all simulation runs (**Supplementary Figures S5**–**S7**). It is worth to mention that, in many simulation cross-validation combinations, no genetic variance could be attributed to failed allele calls; hence, here only the intercept of the model contributed to the prediction (**Supplementary Figures S5**–**S7**).

## 4 Discussion

Utilizing data from three populations in two important crops, we show that failed allele calls can be informative to identify valuable genotype-trait associations in the context of genomic prediction. While the marker number was considerably decreased with failed allele calls compared to standard SNPs, the prediction accuracy was comparable. We developed two alternative pipelines to distinguish failed allele calls with a genuine biological cause from random technical errors. The markers obtained from those two pipelines yielded similar prediction accuracies compared to standard SNPs and to all failed allele calls despite a lower marker density. Therefore, regarding prediction accuracy in genomic prediction, there is no necessity for additional analysis of failed allele calls. Nevertheless, the two pipelines provided enhance the confidence that these failed allele calls arise from a non-random event, possibly attributable to a biological reason. The combinations of the different marker sets did not improve the prediction accuracy, which is likely due to the highly redundant estimation of genomic relationship. However, in cases where failed calls are caused by deletions that are not in LD with neighboring SNPs, it is plausible that they could contribute to improved trait prediction, just as they have been shown to do for QTL analysis [e.g., Gabur et al. (2018); Gabur et al. (2019)].

In both datasets investigated here, failed allele calls were very useful in identifying population structure and relationship, indicating a high relevance of presence–absence variation for population differentiation. Due to different marker filtering and distance calculation, the PCA and the clustering yielded different results in canola than in a previous study using the same dataset (Jan et al., 2016). Interestingly, the failed allele calls were more effective at the identification of present Flint and Dent maize material based on clustering. Sun et al. (2018) and Beló et al. (2010) revealed strong differences between genetically distant maize genotypes in the frequency of copy number variations. Furthermore, in both datasets, one of the two pools had higher average numbers of failed allele calls per line, which can also be observed with the two methods described to filter failed allele calls. This indicates a role of structural variation events underlying failed SNP calls in subpopulation (Gabur et al., 2018) or pool development.

There are several pipelines to detect copy number variations from SNP arrays relying on light intensity signals generated during a single base extension (Colella et al., 2007; Wang et al., 2007; Greenman et al., 2010; Xu et al., 2014; Grandke et al., 2017). However, in case of zero light signal, these pipelines cannot distinguish a genomic deletion from a technically failed allele call. Gabur et al. (2018) provide an alternate strategy to reliably identify genomic deletions using SNP array data. They used segregation patterns of failed allele calls in a nested association mapping population of *Brassica napus* to validate real deletions from technical artifacts of the SNP arrays. Several studies implemented this pipeline to filter and use large numbers of failed allele calls (Gabur et al., 2018; Gabur et al., 2020; Vollrath et al., 2021a; Vollrath et al., 2021b), which are normally removed from downstream analyses by a standard filtering process. However, the pipeline described in those studies cannot be applied in the present study since it relies on deviations from expected allele frequencies in segregating families, whereas the populations investigated here are genetically diverse breeding populations. Therefore, we used pool assignment and LD to filter failed allele calls. These two approaches can be applied to a wider range of populations as they do not need clear family structures while being simple and straightforward to implement. In canola, these two alternative methods delivered similar results: 1,989 failed allele calls filtered based on pool specificity and 1,084 failed allele calls filtered *via* analysis of LD. A pipeline to place markers with unknown chromosomal positions based on LD accurately placed 5,920 out of 21,251 unplaced markers (Yadav et al., 2021). Here with the LD-based filtering method, marker alleles are filtered rather than unplaced markers. The key advantage is that, rather than setting an arbitrary threshold, LD between markers on the same chromosome is used to set a dynamic threshold. Generally, the two pipelines that we developed consider any non-random cause for the allele call failure; however, they cannot classify the cause. While the cause for the allele call failure can have high importance in the detection of major QTL and causal genes, for genomic prediction of quantitative traits, the cause is less relevant as a single marker usually has only a small effect on the prediction (Tayeh et al., 2015; van Binsbergen et al., 2015; Werner et al., 2018a; Werner et al., 2018b; e Sousa et al., 2019).

With the advancements in genotyping technology and the decreasing costs associated with it, genotyping by sequencing (GBS) has emerged as a promising alternative to SNP arrays for genotyping breeding populations (Poland and Rife, 2012; Kim et al., 2016; Chung et al., 2017). Unlike the closed architecture of SNP arrays, which typically only allows the identification of two alleles, GBS has the added advantage of detecting other variants, such as small deletions (Poland and Rife, 2012). This capability offers a potential solution to the aforementioned limitations by directly identifying the true variant at a given locus.

In the canola analysis, the genomic prediction accuracy based on all marker sets roughly corresponded to the original results of Jan et al. (2016). However, for all traits, a small improvement in prediction accuracy could be observed. Compared to Jan et al. (2016), we filtered for SNP markers with a fixed position on the reference genome Express 617 (Lee et al., 2020). Furthermore, we applied a different filtering method for allelic diversity; these together resulted in an additional 2,799 markers. The prediction accuracy across traits and marker sets generally did not deviate considerably from prediction accuracies reported in previous studies, although minor differences can be observed in field emergence and glucosinolate content (Würschum et al., 2014; Jan et al., 2016; Werner et al., 2018a; Werner et al., 2018b; Knoch et al., 2021).

In the maize analysis, the genomic prediction accuracy obtained from all marker sets corresponded to the original results of Lehermeier et al. (2014). The differences can be attributed to the considerably different cross-validation scheme that we used in comparison with the previous study. Furthermore, the different filtering, especially for allelic diversity, resulted in 5,508 more markers compared to the original publication. The accuracies were generally higher than in the canola analysis. As seen in the high prediction accuracies reported in other studies of hybrid prediction in maize (Technow et al., 2012; Crossa et al., 2014; Technow et al., 2014; Millet et al., 2019), we also observed generally high prediction accuracies for all traits and marker sets. Interestingly, the prediction accuracies varied between Flint and Dent datasets. For the traits DtSILK and DtTAS, the prediction accuracy was higher in the test crosses with Dent maternal lines than in the hybrids with Flint maternal lines. Moreover, the two models implemented in a frequentist framework, i.e., GBLUP and EGBLUP, delivered poorer predictions than the remaining models for all traits with the Dent test crosses. This behavior was not observed in the Flint or canola test crosses.

Importantly, predictions based on one of the three marker sets including failed allele calls always gave prediction accuracies competitive with standard SNP-based predictions. The simulation study indicates that this prediction accuracy seems to be not occurring by chance as the randomly sampled failed allele calls in the simulations resulted in a prediction accuracy close to zero. While failed allele calls were observed to be equally predictive as standard SNPs, it is essential to note that this might not directly translate to the entire germplasm of the given crop. This is because SNP arrays usually undergo thorough validation before being released for use. Of course, SNPs are influenced and linked to structural variations like deletions and insertions (Hinds et al., 2006; McCarroll et al., 2006; Redon et al., 2006; Gabur et al., 2018). Our analyses indicated that at least a proportion of the failed allele calls stem from structural variants. The two hybrid breeding crops maize and canola are known to be highly influenced by structural variants (Schnable et al., 2009; Springer et al., 2009; Beló et al., 2010; Lai et al., 2010; Swanson-Wagner et al., 2010; He et al., 2017; Samans et al., 2017; Hurgobin et al., 2018; Sun et al., 2018; Chawla et al., 2021). Furthermore, it is well known that structural variations like deletions, insertions, or inversions can be associated with agronomical traits (Würschum et al., 2015; Gabur et al., 2018; Gabur et al., 2019; Schiessl et al., 2019; Gabur et al., 2020; Vollrath et al., 2021a; Vollrath et al., 2021b) and differential gene expression (Shen et al., 2006; McHale et al., 2012; Tan et al., 2012; Chiang et al., 2017; Alonge et al., 2020). Hence, it can be assumed that the inclusion of SV data can improve the genomic prediction accuracy for some traits in crops; however, just like what is shown here, an improvement is not consistently observed (Hay et al., 2018; Lyra et al., 2019; Knoch et al., 2021). Furthermore, in cattle, only a marginal improvement in prediction accuracy was observed for important milk traits when accounting for structural variations from whole-genome sequencing (Chen et al., 2021).

Although machine learning has promising capabilities in genomic prediction (Montesinos-López et al., 2018; Pérez-Enciso and Zingaretti, 2019; Montesinos-López et al., 2021; Montesinos López et al., 2022), with encouraging results in human (Bellot et al., 2018; Lello et al., 2018), animal (González-Recio et al., 2010; Long et al., 2010; Gianola et al., 2011), and plant research (Heslot et al., 2012; Crossa et al., 2017; Montesinos-López et al., 2018; Azodi et al., 2019; Bayer et al., 2021), we failed to observe any fundamental advantage of two tested machine learning algorithms for any trait, population, or marker set. In contrast to the findings of González-Recio et al. (2010); Li et al. (2018), and Abdollahi-Arpanahi et al. (2020), we did not observe a competitive prediction accuracy of the boosting algorithm XGB in comparison to the other prediction models for 14 out of the 17 examined traits. This corresponds to the findings of Perez et al. (2022). Hyperparameter tuning is crucial for machine learning (Pérez-Enciso and Zingaretti, 2019; Zingaretti et al., 2020; Montesinos López et al., 2022). In this study, we applied a Bayesian hyperparameter optimization which, based on a given set of hyperparameter starting values, optimizes the hyperparameters sequentially with the objective of reducing the mean squared prediction error. It is possible that this optimization algorithm becomes obstructed in a local optimum, resulting in low prediction accuracies. However, it seems unrealistic that this would have occurred in every cross-validation run. Alternatively, the size of the training datasets that we used might be too small for machine learning models, which usually cope with *n* > *p* problems (Azodi et al., 2019).

Incomplete LD between markers and QTL can lead to apparent or phantom epistasis. This can cause statistically significant marker interactions in association studies (Wood et al., 2014; de los Campos et al., 2019) and improved prediction accuracies with models considering epistasis (Schrauf et al., 2020). For predictions using only one of the failed marker sets, we need to assume the occurrence of considerable phantom epistasis due to the considerably lower marker number, which tends to result in lower LD between markers and QTL (Wood et al., 2014; de los Campos et al., 2019). For this reason, we extended the prediction portfolio from GBLUP and Bayesian LASSO to also include EGLUP and RKHS regression for explicit modeling of epistasis and the two machine learning methods SVM and XGB for modeling of nonlinear effects. However, models considering epistasis or nonlinear effects did not consistently outperform simple GBLUP or Bayesian LASSO in any of the failed marker sets. A possible explanation could be that, despite the reduced marker density, a sufficient proportion of QTL can nevertheless be covered by these markers. Indeed marker density can often be reduced without a considerable loss of prediction accuracy (de Roos et al., 2009; Zhang et al., 2019; Kriaridou et al., 2020). Besides co-segregation or LD between markers and QTL, another important factor impacting genomic prediction is the accurate estimation of relationship (Habier et al., 2010; Daetwyler et al., 2013; Habier et al., 2013). In fact, accurate pedigree information can already yield prediction accuracies that are comparable to predictions based on genomic information (Burgueño et al., 2012; Crossa et al., 2014; Deomano et al., 2020). The high correlations between relationship coefficients obtained from SNP markers and the three marker sets from failed allele calls show that information about failure of allele calls can be a good estimate for relationships between genotypes. The correlations between SNP markers and the three respective marker sets from failed allele calls were considerably lower in canola than in maize; however, losses in prediction accuracies were on a similar level in both species. Since SNPs are still only a fraction of all genetic information present on the genome, even SNPs are only able to “sample” a true relationship (Goddard et al., 2011), which could explain the comparable loss of prediction accuracy between the two datasets. However, the high correlation between relationship coefficients also explains the lack of gain in prediction accuracy, indicating that the information added by the failed SNP calls is at least partly redundant. In populations in Hardy–Weinberg equilibrium, this redundant information likely corresponds to SNPs within older deletions that are in LD with surrounding SNPs, whereas more recent structural variants leading to deletions (and failed SNP calls) are not always in LD with redundant SNPs and more likely to contribute additional information to predictions.

While we only observed marginal to no increases in prediction accuracy based on combinations of SNPs with failed marker calls, they may be especially beneficial in the context of association studies, where it has been shown that previously undetected QTL can be identified with the inclusion of failed SNP allele calls (Gabur et al., 2018). Furthermore, the analytical approaches applied here are straightforward to implement with no additional cost.

## 5 Conclusion

Our study confirms that failed allele calls from SNP array data can be highly predictive for agronomical traits in canola and maize. Based on population structure (pool specificity) and LD, we were able to distinguish random errors from systematic allele call failure, enabling the filtering of presence–absence marker data representing deletions with potential impacts on traits. In all examined traits and datasets, genomic prediction using presence–absence markers filtered from failed SNP calls was nearly as accurate as SNP-based prediction. This is likely due to the following: (a) capture of previously overlooked genomic regions, (b) accurate estimation of relationships (similar to SNP-based relationship), and (c) capture of dominance effects caused by deletions which differentiate between heterotic pools in hybrid breeding. However, prediction accuracy did not improve when combining SNP information with failed allele calls, which can be attributed to the high redundancy between estimates of genomic relationship. Nevertheless, we recommend the inclusion of information of allele call failure into genomic prediction, as it adds information that is potentially highly predictive for agronomic traits not always in LD with neighboring SNPs and is available to plant breeders using SNP array datasets for genotyping at no additional cost.

## Data availability statement

The original contributions presented in the study are included in the article/**Supplementary Material**. Further inquiries can be directed to the corresponding author.

## Author contributions

SW and RS designed the study. SW conceived the analysis, MF developed the software for LD calculation and supervised the statistical analysis. LE assisted with the statistical analysis. SW wrote the manuscript. RS, LH, and HC revised the manuscript. All authors contributed to the article and approved the submitted version.
