# Supplementary material for: Accurate prediction of quantitative traits with failed SNP calls in canola and maize
Source: Front Plant Sci. 2023 Oct 23;14:1221750. doi: 10.3389/fpls.2023.1221750 (PMC10627008; doi:10.3389/fpls.2023.1221750)
Supplement: Supplementary file 1 [file DataSheet_1.docx]

Supplementary Material

**Accurate prediction of quantitative traits with failed SNP calls in canola and maize**

Sven E. Weber^1*^, Harmeet Singh Chawla^2^, Lennard Ehrig^1^, Lee T. Hickey^3^, Matthias Frisch^4^, Rod J. Snowdon^1^

*** Correspondence: Sven E. Weber**: Sven.E.Weber@agrar.uni-giessen.de

## Supplementary Figures

**
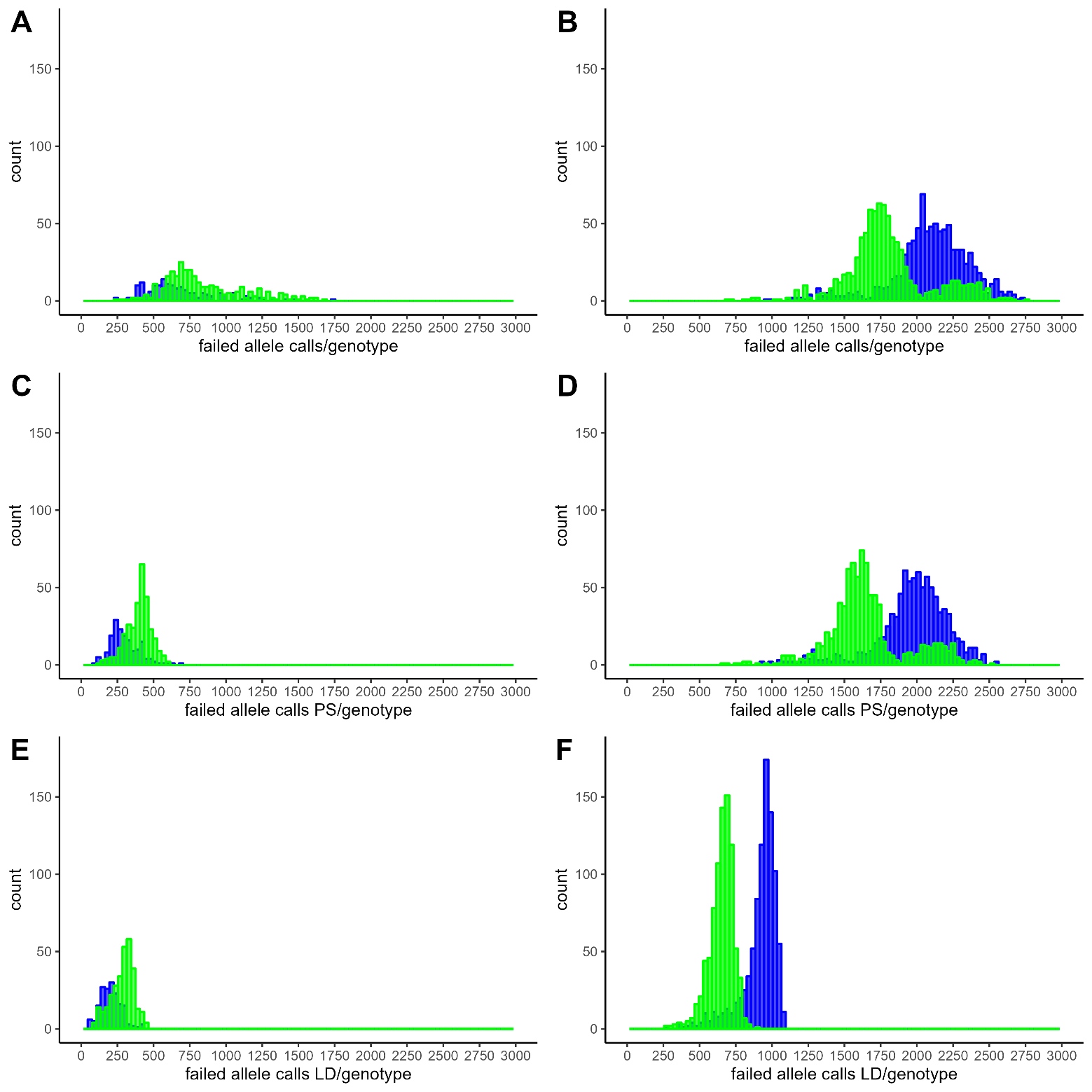
**

Figure S1 Histograms of number of failed allele calls per genotype (A, B) failed allele calls filtered by pool specificity (C, D) and failed allele calls filtered by LD (E, F) in canola (A, C, E) and maize (B, D, F). In canola, the color blue represents pool A and lime pool B. In maize, blue represents the flint pool and lime the dent pool

**
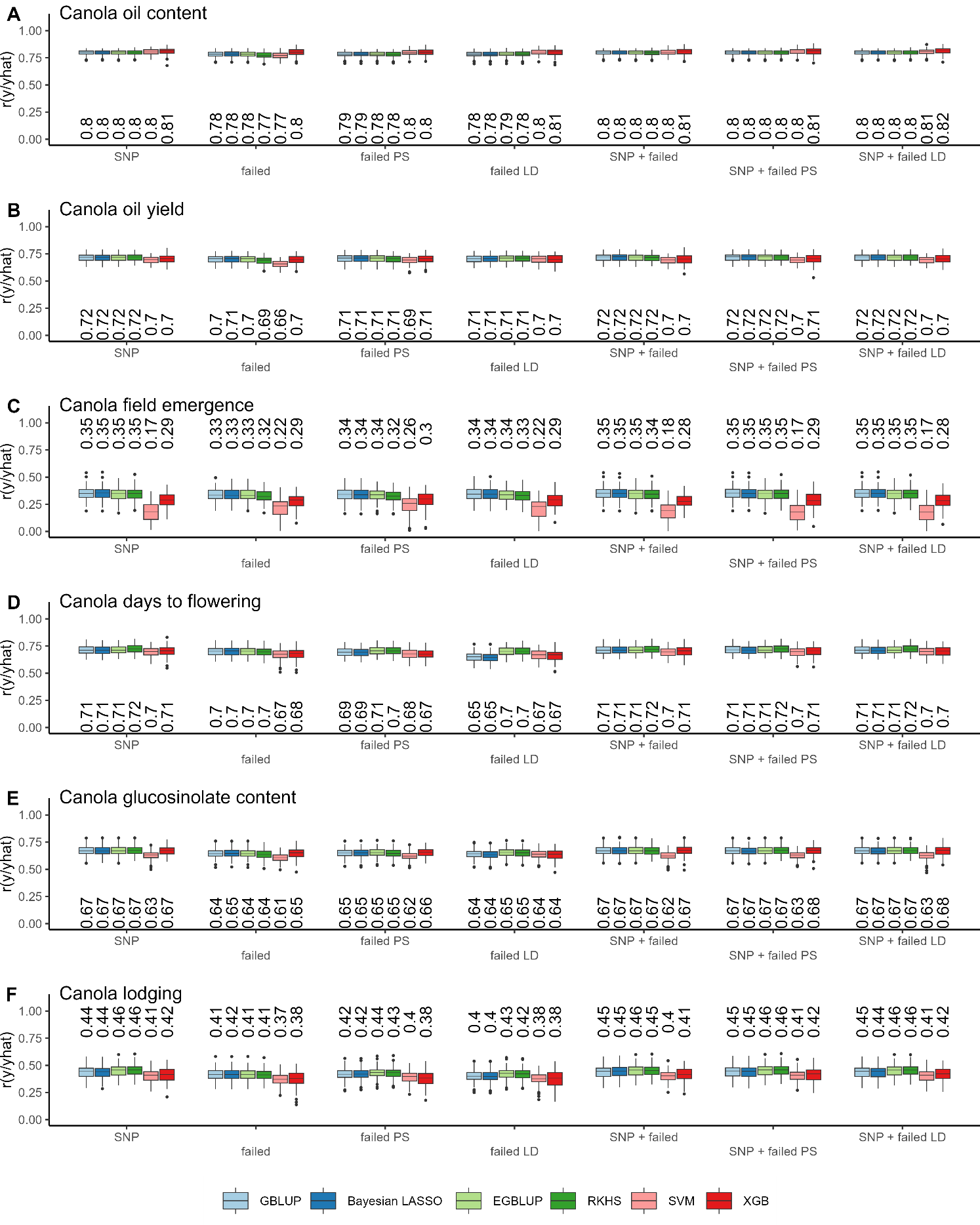
**

**Figure S2** Prediction accuracy (r) based on standard SNPs, failed SNP calls (failed), failed SNP calls filtered by pool specificity (failed PS) and failed SNP calls filtered by LD (failed LD) as well as their combination with GBLUP (light blue), Bayesian Lasso (dark blue), EGBLUP (light green), RKHS (dark green), SVM (pink) and XGB (red). In canola traits: oil content (**A**), oil yield (**B**), field emergence (**C**), days to flowering (**D**), glucosinolate content (**E**) and lodging (**F**) . Values above boxplots represent median values across all cross validation runs


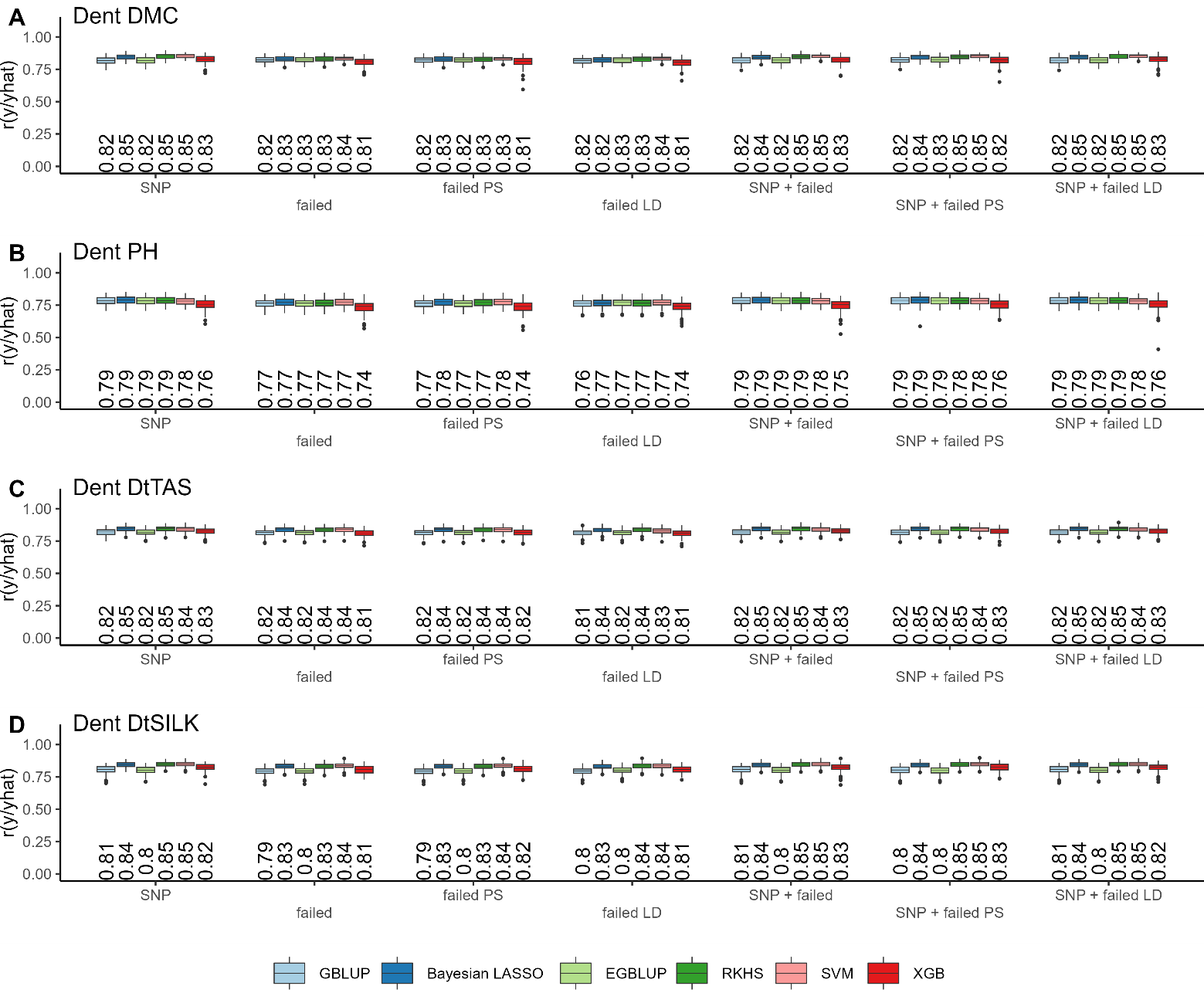
**Figure S3** Prediction accuracy (r) based on standard SNPs, failed SNP calls (failed), failed SNP calls filtered by pool specificity (failed PS) and failed SNP calls filtered by LD (failed LD) as well as their combination with GBLUP (light blue), Bayesian Lasso (dark blue), EGBLUP (light green), RKHS (dark green), SVM (pink) and XGB (red). In maize dent traits: DMY (**A**), DMC (**B**), DtTAS (**C**) and DtSILK (**E**). Values above boxplots represent median values across all cross validation runs


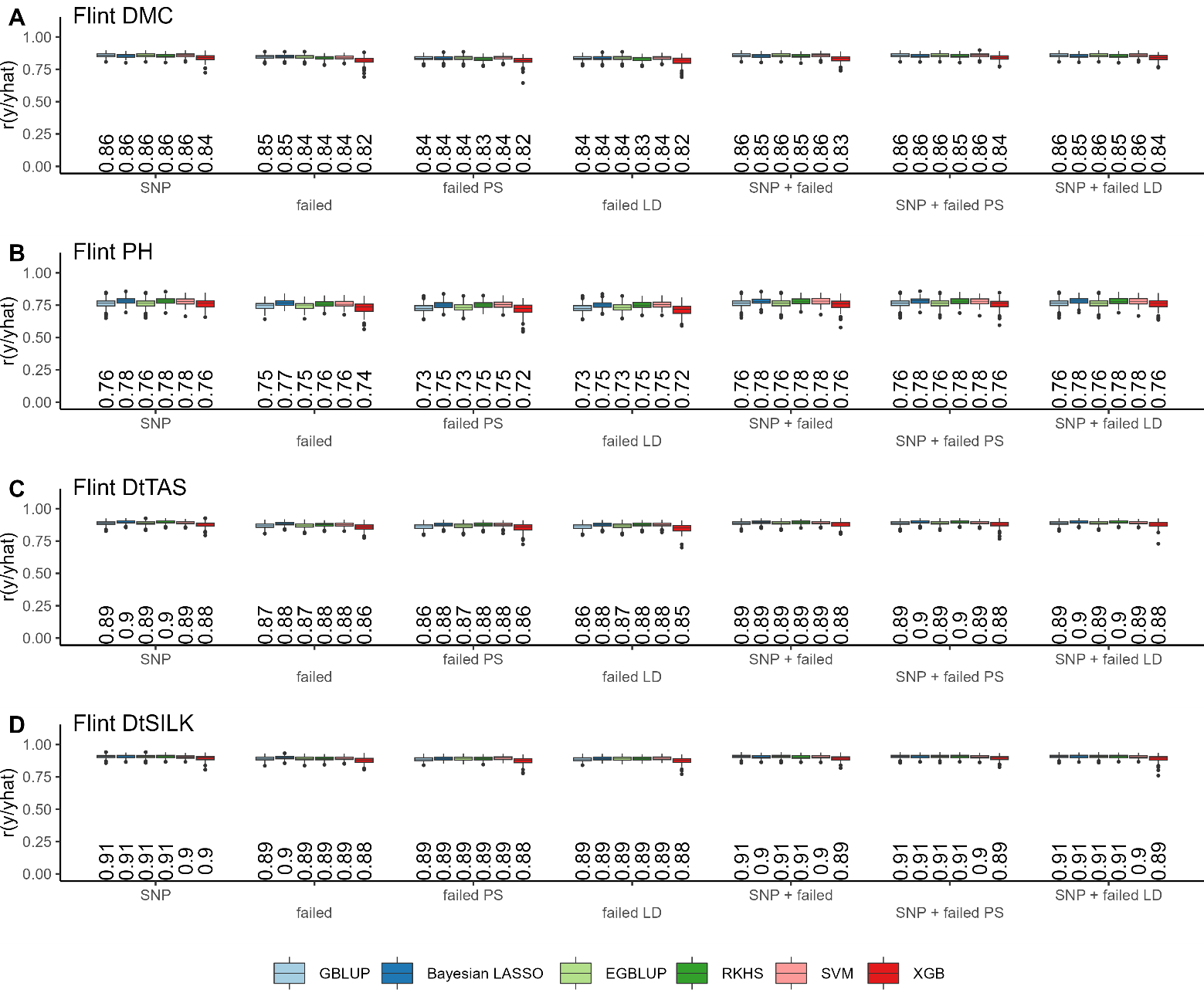


**Figure S4** Prediction accuracy (r) based on SNPs, calls failed SNP calls (failed), failed SNP calls filtered by pool specificity (failed PS) and failed SNP calls filtered by LD (failed LD) as well as their combination with GBLUP (light blue), Bayesian Lasso (dark blue), EGBLUP (light green), RKHS (dark green), SVM (pink) and XGB (red). In maize flint traits: DMY (**A**), DMC (**B**), DtTAS (**C**) and DtSILK (**E**). Values above boxplots represent median values across all cross validation runs


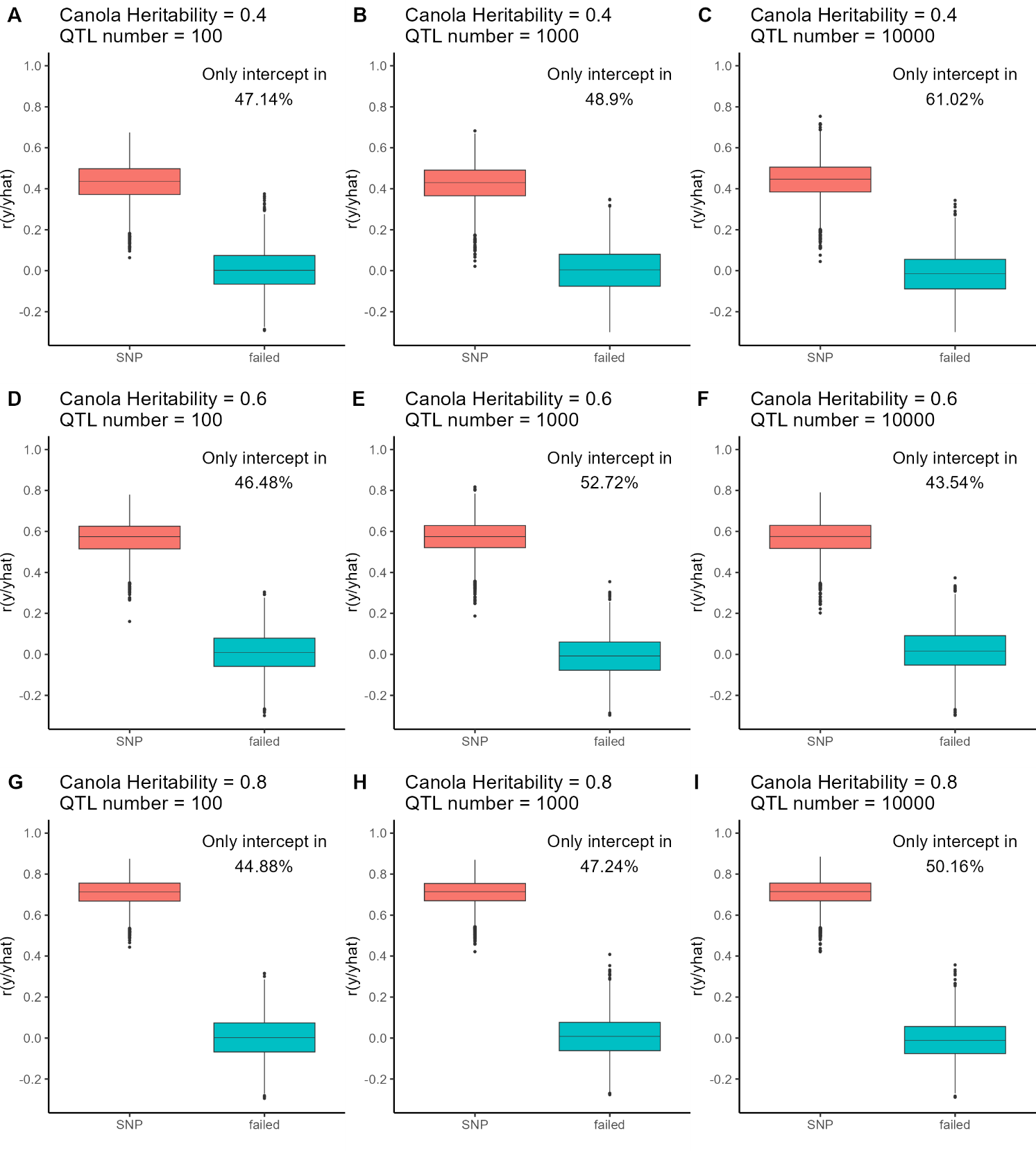


**Figure S5** Results of simulated traits based on the genotypic data of the canola dataset. Prediction accuracy (r) across all simulation and cross-validation runs based on SNPs and a randomly sampled failed SNP calls (failed) with the GBLUP model. **A**, **B**, **C** show traits with a simulated heritability of 0.4. **D**, **E**, **F** show traits with simulated heritability of 0.6. While **G**, **H**, **I** display traits with heritability of 0.8. The number of QTL was 100 for **A**, **D**, **G**, 1,000 for **B**, **E**, **H** and 10,000 for **C**, **F**, **I**.


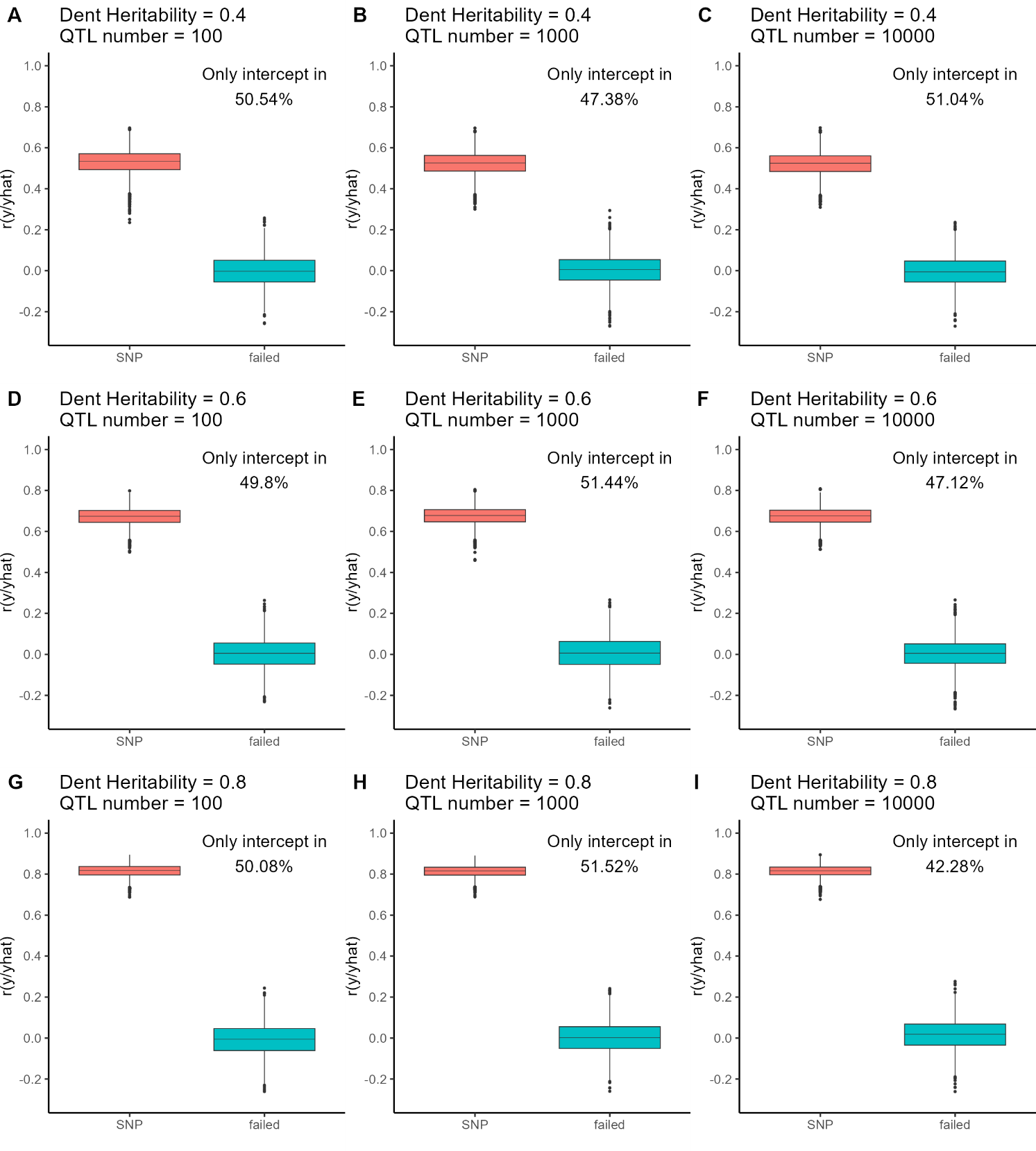


**Figure S6** Results of simulated traits based on the genotypic data of the maize dent dataset. Prediction accuracy (r) across all simulation and cross-validation runs based on SNPs and a randomly sampled failed SNP calls (failed) with the GBLUP model. **A**, **B**, **C** show traits with a simulated heritability of 0.4. **D**, **E**, **F** show traits with simulated heritability of 0.6. While **G**, **H**, **I** display traits with heritability of 0.8. The number of QTL was 100 for **A**, **D**, **G**, 1,000 for **B**, **E**, **H** and 10,000 for **C**, **F**, **I**.


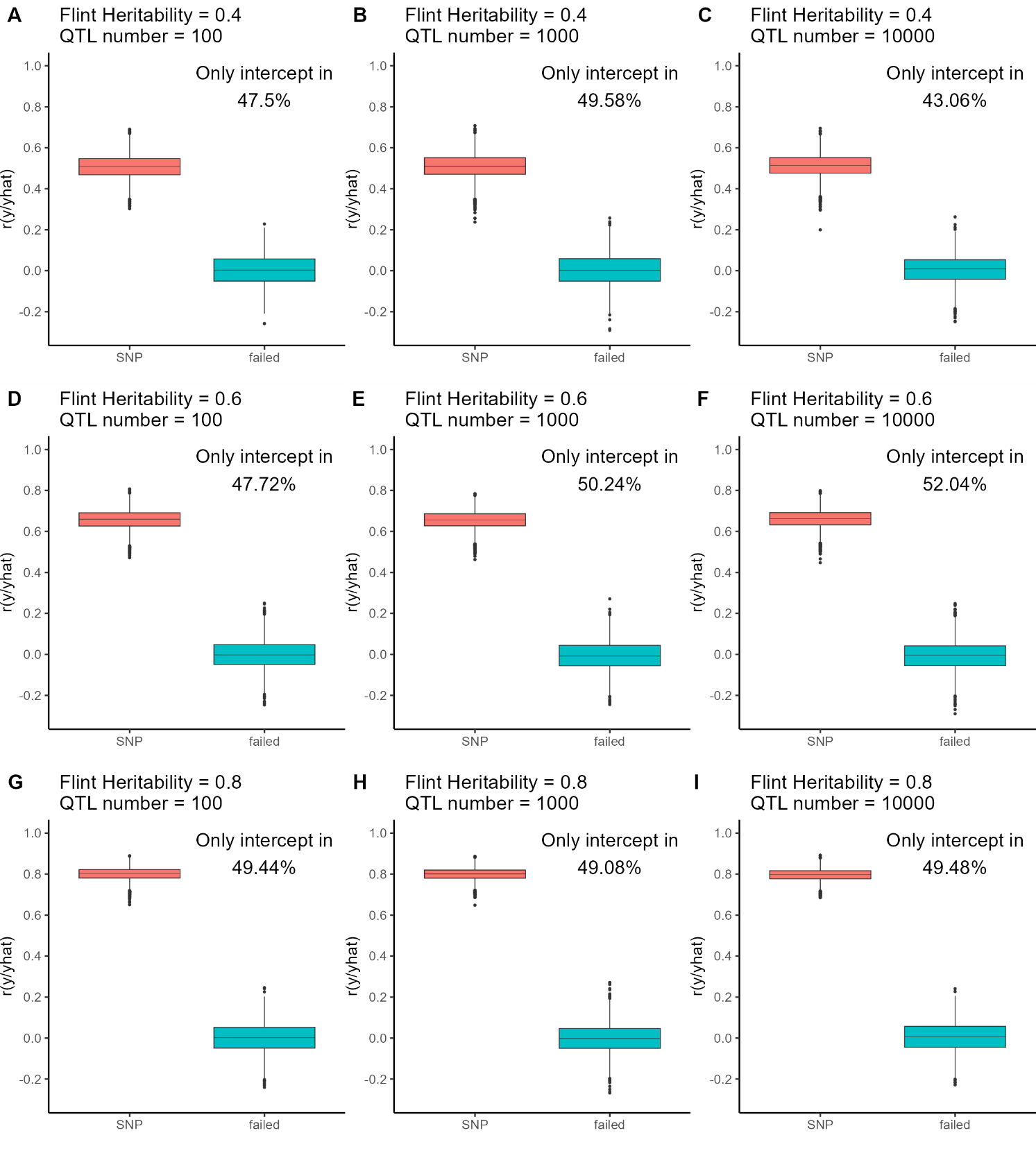


**Figure S7** Results of simulated traits based on the genotypic data of the maize flint dataset. Prediction accuracy (r) across all simulation and cross-validation runs based on SNPs and a randomly sampled failed SNP calls (failed) with the GBLUP model. **A**, **B**, **C** show traits with a simulated heritability of 0.4. **D**, **E**, **F** show traits with simulated heritability of 0.6. While **G**, **H**, **I** display traits with heritability of 0.8. The number of QTL was 100 for **A**, **D**, **G**, 1,000 for **B**, **E**, **H** and 10,000 for **C**, **F**, **I**.
